# Retained fetal bones after second-trimester dilation and curettage (D&C): report of two cases and review of literature

**DOI:** 10.1097/RC9.0000000000000218

**Published:** 2026-02-10

**Authors:** Daudi Gidion, Mekyus E. Sanga, Shweta Jaiswal, Naomi Sanga, Zainab Fidaal, Lynn Moshi

**Affiliations:** a The Aga Khan University, Department of Obstetrics and Gynaecology, Dar Es Salaam, United Republic of Tanzania; b The Aga Khan University, Department of Radiology, Dar Es Salaam, United Republic of Tanzania

**Keywords:** case report, hysteroscopy, post-abortion care, secondary infertility

## Abstract

**Introduction:**

Retained fetal bones are a rare complication following second-trimester dilation and curettage (D&C). This condition can lead to chronic endometrial inflammation and secondary infertility, often presenting years later. Missed diagnoses are especially common in low-resource settings due to limited follow-up and diagnostic capacity.

**Presentation of cases:**

We present two cases of women with histories of second-trimester pregnancy loss managed with D&C, later diagnosed with retained intrauterine fetal bones.

*Case 1*: A 30-year-old woman, Gravida 5, Para 2, presented 2 weeks post-abortion with ultrasound findings of hyperechogenic structures in the endometrial cavity. Hysteroscopy confirmed and removed multiple bone fragments.

*Case 2:* A 29-year-old woman, Gravida 3, Para 0, presented with 6 years of secondary infertility. Ultrasound revealed echogenic material in the uterine cavity. Operative hysteroscopy removed calcified fetal remnants. Both patients had successful hysteroscopic removal and resumed normal menses postoperatively.

**Discussion:**

Retained fetal bones act as intrauterine foreign bodies, causing secondary infertility through mechanical obstruction, chronic inflammation, and impaired endometrial receptivity. Delayed diagnosis is common due to nonspecific symptoms and limited access to imaging. These cases highlight systemic gaps in post-abortion care, including inadequate follow-up and provider awareness.

**Conclusion:**

These cases highlight the need to consider retained fetal bones in women with unexplained infertility and a prior history of second-trimester abortion. Routine post-procedure imaging and structured follow-up protocols are crucial for timely diagnosis and management, especially in resource-limited settings.

## Introduction

Retained intrauterine fetal bone is an uncommon complication following second-trimester abortion, particularly when performed via dilation and curettage (D&C). Although rare, this condition has been increasingly reported in the literature as a cause of abnormal uterine bleeding and secondary infertility, often years after the initial event. Most cases go undetected due to nonspecific symptoms and the lack of structured post-abortion follow-up, especially in low-resource settings[1].

The true incidence of retained fetal bones remains unknown, largely due to underreporting and diagnostic delays. Case series have shown that these fragments can persist in the endometrial cavity for years, often mimicking intrauterine devices on ultrasound[2]. Histologically, retained fetal bones are non-viable and lack trophoblastic tissue. However, their presence triggers chronic endometrial inflammation, impairs endometrial receptivity, and disrupts implantation, ultimately causing secondary infertility[1].

HIGHLIGHTS

- Retained intrauterine fetal bones are an uncommon but underrecognized cause of secondary infertility following second-trimester abortion.
- Diagnosis can be delayed for years due to non-specific symptoms and lack of structured post-abortion follow-up, particularly in low-resource settings.
- Transvaginal ultrasound may suggest the diagnosis, but hysteroscopy with histopathological confirmation remains the gold standard.
- Hysteroscopic removal of bone fragments is curative in most cases and can restore fertility, even after prolonged retention.
- Early imaging after second-trimester abortion can prevent long-term reproductive sequelae.

In high-income settings, advances in imaging and minimally invasive hysteroscopic techniques have improved detection and management, but these are often unavailable or underutilized in lower-level health facilities[3]. Moreover, systemic gaps in post-abortion care, such as lack of routine follow-up, limited training on mid-trimester complications, and fragmented referral pathways, contribute to missed diagnoses and long-term reproductive sequelae^[^3–5^]^.

This article presents two cases of retained fetal bones following second-trimester D&C. Through these cases and a review of the literature, we aim to highlight key diagnostic pitfalls and underscore the need for proper post-abortion follow-up protocols, particularly in decentralized care settings. This work has been prepared and reported in accordance with the SCARE criteria[6].

## Presentation of cases

### Case 1

A 30-year-old woman, Gravida 5, Para 2, Living 2, with hypothyroidism on levothyroxine, presented at 17 weeks of gestation, confirmed by first-trimester ultrasound, with a 2-day history of per vaginal bleeding and lower abdominal pain. On physical examination, the patient’s vital signs were stable. Speculum examination revealed a healthy cervix with no abnormal discharge or visible lesions. Bimanual examination demonstrated a normally sized, nontender uterus with no palpable adnexal masses. There were no signs of infection or pelvic inflammation.

She was diagnosed with a missed abortion and underwent dilation and curettage. Histopathological examination revealed fetal parts, including bone, umbilical cord, and chorionic villi. Microscopy showed hyalinized villi with focal necrosis and mixed inflammatory infiltrates, consistent with products of conception and possible subacute villitis.

**Figure 1.**
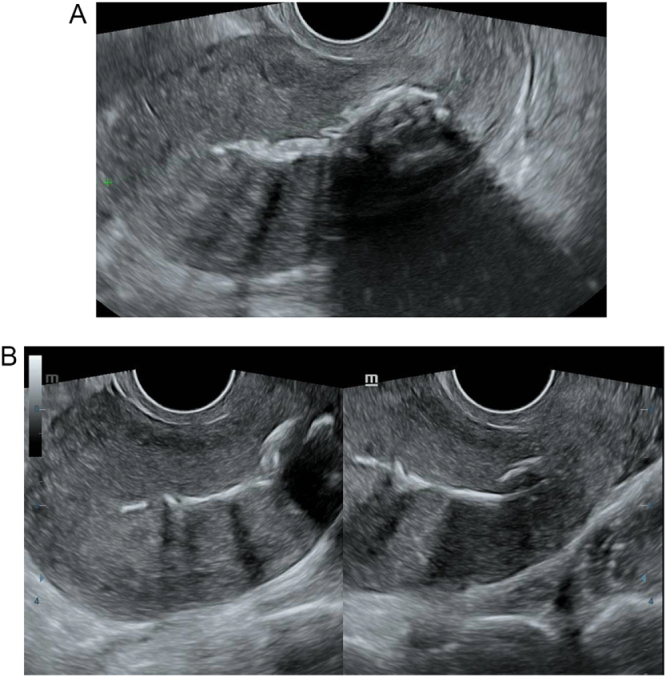
(A) Sagittal transvaginal ultrasound showing hyperechogenic structures within the endometrial cavity with posterior acoustic shadowing, consistent with retained fetal bones. (B) Transvaginal ultrasound transverse views showing hyperechogenic structures within the endometrial cavity with posterior acoustic shadowing, consistent with retained fetal bones.

**Figure 2.**
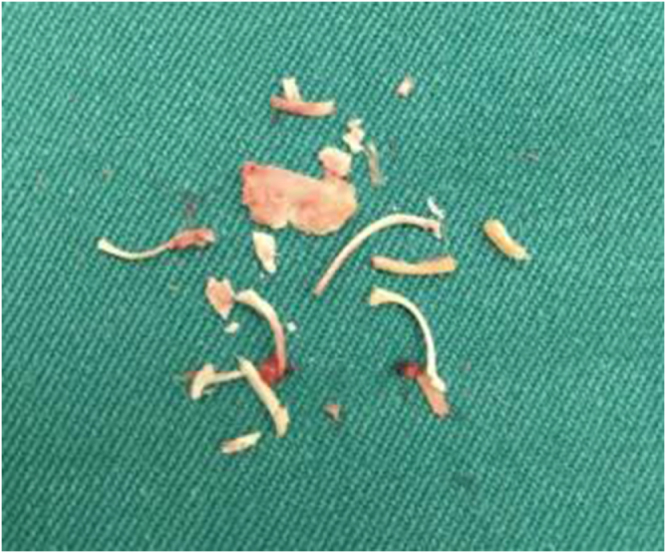
Hysteroscopic retrieval showing multiple devitalized bone fragments removed from the endometrial cavity.

The immediate postoperative period was uneventful, with no further bleeding or passage of tissue. Two weeks later, on follow-up, a transvaginal ultrasound (TVUS) revealed multiple hyperechogenic structures with posterior acoustic shadowing within the endometrial cavity, consistent with retained fetal bones (Fig. 1). Serum human chorionic gonadotropin (β-hCG) was negative. She underwent operative hysteroscopy under general anesthesia. Multiple bone fragments were visualized and successfully removed (extracted specimens shown in Fig. 2). The procedure was uneventful. At 2-month follow-up, she remained asymptomatic with regular menses and no immediate plans to conceive therefore an intrauterine contraceptive device was inserted.

### Case 2

A 29-year-old woman, Gravida 3, Para 0, presented with secondary infertility for 6 years. She had a miscarriage at 20 weeks of gestation in 2019, managed with D&C at a peripheral facility. No postoperative complications were documented at that time.

**Figure 3.**
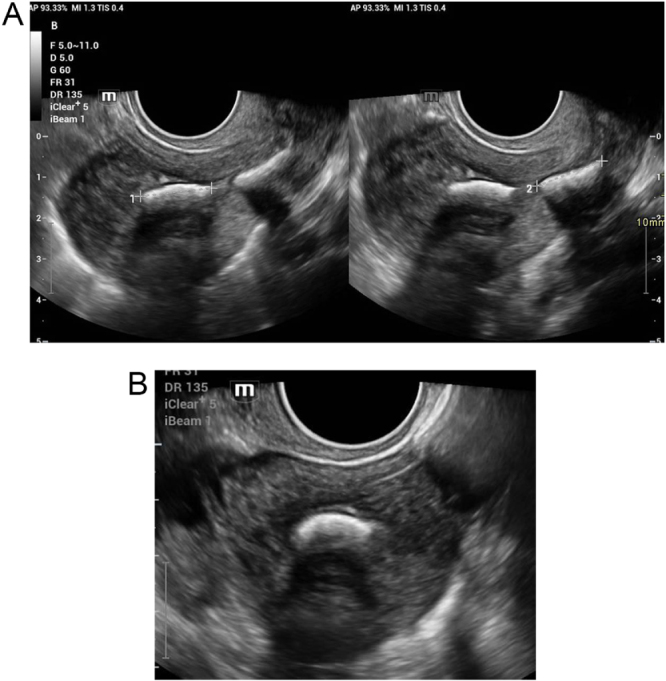
(A). Transvaginal ultrasound sagittal views demonstrating linear hyperechoic intrauterine structures, suggestive of retained fetal bones/endometrial calcifications prior to hysteroscopic confirmation. (B). Transvaginal ultrasound transverse views demonstrating linear hyperechoic intrauterine structures, suggestive of retained fetal bones/endometrial calcifications prior to hysteroscopic confirmation.

She reported regular 28–30-day cycles, lasting 3–4 days, with normal flow. She had no dysmenorrhoea, intermenstrual bleeding, or postcoital bleeding. There was no history of dyspareunia, abnormal vaginal discharge, or pelvic infections. She had never used contraception. Clinical examination showed a healthy vulva and vagina. Speculum examination revealed no discharge, cervical erosion, or foreign body. On bimanual palpation, the uterus was of normal size and nontender. There was no adnexal fullness or tenderness noted. The pelvic exam was essentially unremarkable. Baseline infertility evaluation was initiated. TVUS revealed echogenic intrauterine material, raising suspicion for retained fetal bone fragments (Fig. 3). Further testing, including hormonal profile (Follicle stimulating hormone (FSH), Lutenizing Hormone (LH), prolactin, Thyroid stimulating hormone (TSH), and oestradiol), was within normal limits. Tubal patency assessment was deferred in light of the intrauterine findings. The partner’s semen analysis was normal.

**Figure 4.**
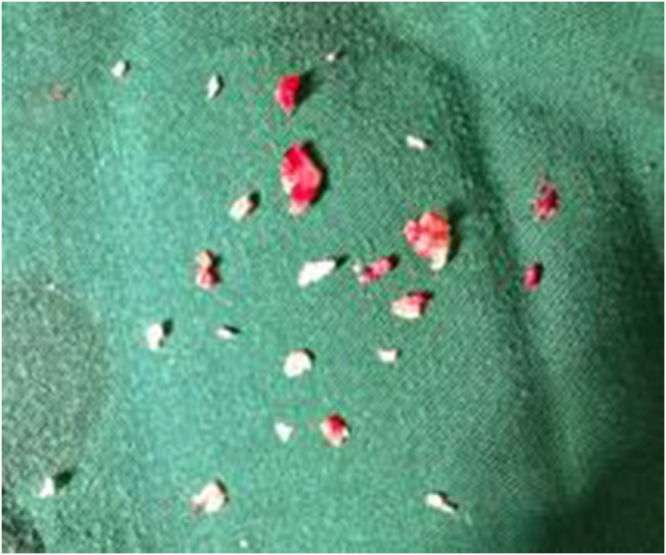
Hysteroscopic retrieval showing bone fragments removed from the endometrial cavity.

She underwent operative hysteroscopy under general anesthesia. Multiple bony fragments were removed using hysteroscopic forceps (extracted specimens shown in Fig. 4). No intraoperative or postoperative complications occurred. Histopathology confirmed devitalized fetal bone with no trophoblastic elements. At the 2-month follow-up, she was asymptomatic and had resumed regular menstrual cycles, and at the 4-month follow-up, she had a natural conception. A comparison summary of the two cases is presented in Table 1.

**Table 1:** Comparison of clinical and diagnostic features in two cases of retained intrauterine fetal bones

Feature	Case 1	Case 2
Age	30 years	29 years
Gravida/para	P2+3	P0+2
Time to diagnosis	2 weeks post-D&C	6 years after D&C
Symptoms	None	Secondary infertility
Clinical exam	Normal	Normal
TVUS findings	Echogenic intrauterine debris	Echogenic intrauterine debris
Hysteroscopy findings	Retained bone fragments	Retained bone fragments
Histology	Devitalized bone	Devitalized bone
Outcome	Normal on follow-up	Conceived 4 months after removal

Summary of demographic, clinical, diagnostic, and outcome features in two cases of retained intrauterine fetal bones after second-trimester D&C. Case 1 was diagnosed within weeks, while Case 2 was diagnosed after 6 years during infertility evaluation.

## Discussion

To contextualize these cases, we reviewed published literature on retained fetal bones, with particular focus on diagnostic approaches, clinical implications, and systemic contributors to delayed diagnosis. This discussion synthesizes existing evidence and highlights areas where clinical practice and post-abortion care protocols may fall short, especially in resource-limited settings.

Although the exact incidence of retained fetal bones is unknown due to underreporting, case series suggest it is not uncommon among women with prior second-trimester terminations. A systematic review of 75 cases by Agolli *et al*[7] revealed that 60% had a history of second-trimester D&C, with many presenting years later with infertility. The diagnostic sensitivity of TVUS varies with operator skill and fragment characteristics. While echogenic intrauterine material with posterior acoustic shadowing is suggestive, it is not pathognomonic. Fragments are frequently missed at facilities lacking high-resolution imaging or experienced radiologists^[^8–10^]^. This reinforces the value of hysteroscopy for definitive diagnosis.

Retained fetal bones act as intrauterine foreign bodies, mimicking the contraceptive effect of an IUCD. They induce a sterile inflammatory response characterized by prostaglandin release, stromal edema, and leukocyte infiltration. These changes, combined with mechanical obstruction, compromise endometrial receptivity and impair embryo implantation, resulting in secondary infertility^[^9,10^]^. Additionally, prolonged retention can cause menstrual irregularities, pelvic pain, and dysmenorrhoea^[^11,12^]^.

While TVUS is a useful first-line tool, echogenic intrauterine structures are not specific to retained fetal bones. Differential diagnoses include endometrial calcifications, osseous metaplasia, IUCD fragments, and calcified submucosal fibroids. Hysteroscopy offers direct visualization but still requires histopathological confirmation to exclude other pathologies such as osseous metaplasia or retained products of conception with necrosis. In our cases, the diagnosis was confirmed by histology showing devitalized bone without trophoblastic tissue, ruling out ongoing gestational tissue or malignancy.

Diagnosis is often delayed due to nonspecific symptoms and limited access to advanced imaging modalities in low-resource settings[8]. Hysteroscopic removal of retained fetal bones is the gold standard for both diagnosis and treatment. This minimally invasive approach enables complete extraction of bony fragments, often leading to the restoration of normal menstrual cycles and fertility. In a study from India, 27.7% of women conceived after hysteroscopic retrieval, highlighting the potential for fertility restoration^[^9,12^]^.

The delayed diagnosis in both presented cases underscores systemic gaps in post-abortion care, particularly in low-resource settings. Factors contributing to these gaps include a lack of routine follow-up protocols, limited provider awareness of mid-trimester complications, and inadequate access to diagnostic tools. In Tanzania, for instance, only eight facilities per 100 000 women provide post-abortion care, leading to significant disparities in access[13], and less than 30% of women receive follow-up within 2 weeks of the procedure[13]. A review of Sub-Saharan Africa found that 43% of women needing post-abortion treatment did not receive any care[14].

The retention of fetal bones following second-trimester dilation and curettage (D&C) likely results from incomplete evacuation during the initial procedure^[^1,15^]^. Several factors may contribute to this risk. Inadequate cervical dilation prior to the procedure can limit access to the uterine cavity and increase the likelihood of retained tissue; current guidelines recommend appropriate cervical preparation using osmotic dilators or pharmacological agents before mid-trimester surgical evacuation[16]. Additionally, operator inexperience with mid-trimester procedures has been associated with higher complication rates, as these procedures require specialized training and skill compared to first-trimester evacuations[16]. Limited visualization of the uterine cavity during the procedure and the absence of post-procedure verification of complete evacuation further compound the risk, particularly in resource-limited settings where ultrasound guidance may be unavailable[15]. These factors collectively highlight the need for improved training, adherence to cervical preparation protocols, and systematic post-procedure assessment to reduce the incidence of retained fetal bones.

Both patients presented with a history of second-trimester pregnancy loss managed with D&C. While Case 1 was identified and treated within weeks due to routine follow-up imaging, Case 2 experienced a 6-year delay in diagnosis, only presenting during infertility workup. Despite differing timeframes, both cases were managed effectively through hysteroscopic removal, highlighting the utility of minimally invasive approaches even in delayed presentations.

Similar diagnostic delays of 2–10 years have been reported by Bakhshi *et al* and Agolli *et al*^[^7,12^]^. Case 2 reflects patterns common in low-resource settings, contrasting with Case 1’s early detection through routine imaging. Both cases were asymptomatic, reinforcing the need for structured post-procedural evaluation regardless of symptoms, particularly in settings with inconsistent follow-up.

A limitation of this report is the absence of intraoperative hysteroscopic images, which were not captured due to equipment constraints. However, the ultrasound images and photographs of extracted bone fragments adequately document the clinical findings.

## Conclusion

These cases underscore the importance of considering retained fetal bones in women presenting with unexplained secondary infertility following second-trimester pregnancy loss. While current guidelines do not mandate routine post-procedure imaging, our experience suggests value in structured follow-up protocols.

We recommend clinical reassessment within 2–4 weeks of mid-trimester D&C, with imaging for patients with concerning symptoms or incomplete evacuation risk factors, enhanced provider training on mid-trimester complications and their recognition, and improved access to diagnostic and operative hysteroscopy in resource-limited settings.

## Data Availability

Not applicable.

## References

[1] Reddy LS Jaiswal A Reddy K. Retained Intrauterine Foetal Bone Fragments Causing Secondary Infertility: A Review. Cureus 2023;15:3. 10.7759/cureus.44005 PMC10517090 37746402

[2] World Health Organization (WHO). Abortion care guideline. Geneva: World Health Organization; 2022. Accessed 15 september 2025. Available from: https://srhr.org/abortioncare/

[3] Muga W Ekott M Oladapo OT. Barriers to post-abortion care service provision: A cross-sectional analysis in Burkina Faso, Kenya and Nigeria PLOS Glob Public Health. 2024;4:e0001862. 38452008 10.1371/journal.pgph.0001862 PMC10919639

[4] Paul M Gemzell-Danielsson K Kiggundu C. Barriers and facilitators in the provision of post-abortion care at district level in central Uganda – a qualitative study focusing on task sharing between physicians and midwives. BMC Health Serv Res 2014;14:28. 24447321 10.1186/1472-6963-14-28 PMC3903434

[5] High Impact Practices in Family Planning (HIPs). Postabortion Family Planning: Strengthening the Family Planning Component of Postabortion Care. Washington, DC: HIPs Partnership; 2022. Accessed 12 september 2025. Available from: https://www.fphighimpactpractices.org/briefs/postabortion-family-planning/

[6] Kerwan A Al-Jabir A Mathew G. Revised Surgical Case Report (SCARE) Guideline: An Update for the Age of Artificial Intelligence. Prem J Sci 2025;10:5.

[7] Agolli A Lawrence JA Maskey U. Secondary Infertility due to Fetal Bone Retention A systematic literature review. Sultan Qaboos Univ Med J 2022;22:448–54. 36407692 10.18295/squmj.6.2022.042 PMC9645505

[8] Graham O Cheng LC Parsons JH. The ultrasound diagnosis of retained foetal bones in West African patients complaining of infertility. BJOG 2000;107:122–24. 10645871 10.1111/j.1471-0528.2000.tb11588.x

[9] Gainder S Arora P Dhaliwal LK. Retained Intrauterine Bony Fragments as a Cause of Secondary Infertility in a Tertiary Level Indian Hospital. J Hum Reprod Sci 2018;11:286. 30568360 10.4103/jhrs.JHRS_33_18 PMC6262664

[10] Xiao S Tian Q Xue M. Infertility caused by intrauterine foetal bone retention: a case report. J Med Case Rep 2014;8:177. 24898732 10.1186/1752-1947-8-177 PMC4077231

[11] Cao J Grubb C Khurshid M. Retained foetal bone post-abortion causing infertility. Clin Case Rep 2022;10:e5966. 35769242 10.1002/ccr3.5966 PMC9210129

[12] Bakhshi PS Allahbadia GN Kaur K. Hysteroscopic removal of intrauterine retained fetal bones. Gynecol Surg 2004 2004;1:159–66.

[13] Keogh SC Kimaro G Muganyizi P. Incidence of induced abortion and post-Abortion care in Tanzania. PLoS One 2015;10:e0133933. 26361246 10.1371/journal.pone.0133933 PMC4567065

[14] Bankole A Remez L Owolabi O. From Unsafe to Safe Abortion in Sub-Saharan Africa: Slow but Steady Progress (New York: Guttmacher Institute); 2020. Accessed 16 september 2025. https://www.guttmacher.org/report/from-unsafe-to-safe-abortion-in-subsaharan-africa. doi:10.1363/2020.32446.

[15] Fox MC Krajewski CM. Cervical preparation for second-trimester surgical abortion prior to 20 weeks’ gestation. Contraception 2014;89:75–84. 24331860 10.1016/j.contraception.2013.11.001

[16] Autry AM Hayes EC Jacobson GF. A comparison of medical induction and dilation and evacuation for second-trimester abortion. Am J Obstet Gynecol 2002;187:393–97. 12193931 10.1067/mob.2002.123887

